# Chronic activation of the innate immune system may underlie the metabolic syndrome

**DOI:** 10.1590/S1516-31802001000300008

**Published:** 2001-05-02

**Authors:** Bruce Bartholow Duncan, Maria Inês Schmidt

**Keywords:** Inflammation, Cytokines, Non-insulin dependent diabetes mellitus, Cardiovascular disease, Obesity, Syndrome X, Inflamação, Citocina, Diabetes não insulino dependente, Doença Cardiovascular, Obesidade, Síndrome X

## Abstract

**CONTEXTO:**

The metabolic syndrome is characterized by a clustering, in free-living populations, of cardiovascular and diabetes risk factors generally linked to insulin resistance, obesity and central obesity. Consonant with the well-established inflammatory pathogenesis of atherosclerotic disease, the metabolic syndrome is now being investigated in relation to its inflammatory nature.

**OBJETIVO:**

We present cross-sectional findings demonstrating that markers of inflammation correlate with components of the metabolic syndrome, and prospective findings of the ARIC Study indicating that markers of inflammation and endothelial dys-function predict the development of diabetes mellitus and weight gain in adults. We present biological evidence to suggest that chronic activation of the innate immune system may underlie the metabolic syndrome, characterizing the common soil for the causality of type 2 diabetes mellitus and cardiovascular disease.

**CONCLUSIONS:**

Better understanding of the role of the innate immune system in these diseases may lead to important advances in the prediction and management of diabetes and cardiovascular disease.

## RESUMO

**CONTEXTO:**

A síndrome metabólica se caracteriza por um agrupamento, em populações, de fatores de risco para doenças cardiovasculares e diabetes geralmente ligados à resistência à insulina, à obesidade e à obesidade central. Consonante com a patogênese inflamatória bem-estabelecida da doença aterosclerótica, a síndrome metabólica está agora sendo pesquisada em relação à sua natureza inflamatória.

**OBJETIVO:**

Apresentamos dados transversais demonstrando que marcadores de inflamação se correlacionam com componentes da síndrome metabólica, e dados prospectivos do Estudo ARIC indicando que marcadores de inflamação e de disfunção endotelial predizem o desenvolvimento de *diabetes mellitus* e ganho de peso em adultos. Apresentamos evidências biológicas que sugerem que a ativação crônica do sistema imune inato pode ser a base da síndrome metabólica, caracterizando o solo comum para a causalidade do *diabetes mellitus* tipo 2 e da doença cardiovascular.

**CONCLUSÕES:**

O melhor entendimento do papel do sistema imune inato nessas doenças poderá levar a avanços importantes na predição e manejo do diabetes e da doença cardiovascular.

## INTRODUCTION

Recent years have seen marked changes in our understanding of the risks for and pathogenesis of a series of chronic diseases, among them obesity, diabetes and atherothrombotic cardiovascular disease, which together constitute a major and growing cause of morbidity and mortality in adult populations around the world. The remarkable similarity of the risk factors for coronary heart disease (CHD) and diabetes has stimulated the search for a common pathophysiology for these conditions.^1^ Innumerable investigators are working to understand the clustering of these risk factors in individuals. There are various names for these risk factors: syndrome X,^2^ the insulin resistance syndrome, ^3^ the multiple metabolic syndrome, the plurimetabolic syndrome, or, as suggested by the World Health Organization,^4^ simply the metabolic syndrome. The extent of the clustering has now been demonstrated in community-based populations of diverse ancestries around the world.^3,5–7^ For instance, in the Atherosclerosis Risk in Communities (ARIC) Study, we demonstrated that 30% of the occurrence of five major syndrome abnormalities – hypertension, diabetes, high triglycerides, low high density lipoprotein-C (HDL-C), and high uric acid – aggregated in only 7% of the population. This percentage was three times that expected (11%) by chance alone (P < 0.001).

**Figure 1:**
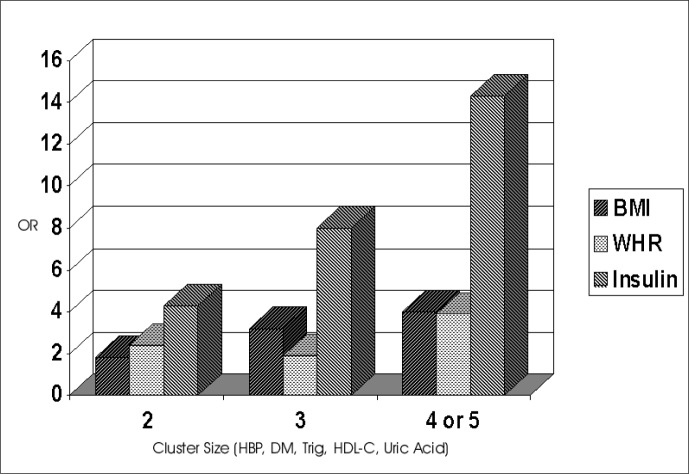
Odds of clustering of the CHD risk factors: hypertension (HBP), diabetes mellitus (DM), high triglycerides (Trig), low high-density lipoprotein cholesterol (HDL-C) and uric acid by levels (5^th^ quintile vs. 1^st^ quintile) of fasting insulin, body mass index (BMI) and waist-hip ratio (WHR), in the Atherosclerosis Risk in Communities Study.

One manner in which to express the tendency for any given factor to aggregate is the odds ratio of the association of that abnormality with the presence of two or more of the additional abnormalities. As seen in Table 1, although variation was present across gender and ethnic groups, all of these five abnormalities were associated with important clustering of the others. Figure 1 shows important associations of the measurements of obesity, central obesity and insulin resistance (fasting insulin) with this clustering. Those with highest (top quintile) values of each of these three factors had considerably greater odds of presenting clusters of two, three, and especially four or more of these factors.^7^ As these analyses were adjusted not only for age, gender, ethnicity and ARIC Study center, but also for the levels of the other two factors, associations expressed in the graph for each are independent of the level of the other two. Additional studies have found the presence of similar clustering in populations of diverse racial and ethnic composition throughout the world.^6^

**Table 1:** Ethnic and gender-specific odds ratios† of an individual presenting a cluster of two or more of the other four metabolic abnormalities (vs. none or only one abnormality), for those presenting a given abnormality. Men and women aged 45 to 64 of the ARIC Baseline Survey, 1987 to 1989

Presenting Abnormality	Odds Ratio			
	WM	WW	AAM	AAW
Hypertension	2.6***	4.0***	2.4***	2.4***
Diabetes	3.4***	7.5***	1.6**	2.0***
High Triglycerides	5.0***	8.8***	5.4***	7.2***
Low HDL-C	3.4***	7.8***	3.1***	2.7***
High Uric Acid	2.6***	7.0***	2.3***	2.2***

* *P < 0.05;*

** *P < 0.01;*

*** *P < 0.001;*

† *Odds ratios obtained from multiple logistic models, controlling for age. The reference exposure category is that without the abnormality listed in the row; the reference end point category is that composed of individuals presenting at most one of the other four abnormalities.; WM = white men, WW = white women, AAM = African-American men, AAW = African-American women; Reproduced^7^ with permission.*

Given the importance of the syndrome, and the recent demonstration of associations between inflammatory markers and syndrome elements, the purpose of this review is to integrate new knowledge concerning the possible interactions of inflammatory mediators with the syndrome.

## BIOLOGICAL EVIDENCE

### Inflammation and Coronary Heart Disease

Impressive evidence has accumulated over the last decade that atherothrombotic cardiovascular disease is based fundamentally on inflammatory pathogenic mechanisms. Russell Ross, one of the main exponents of this hypothesis, recently and elegantly summarized the evidence for the inflammatory nature of atherosclerosis, stating: "The lesions of atherosclerosis represent a series of highly specific cellular and molecular responses that can best be described, in aggregate, as an inflammatory disease".^8^ The initial steps of atherosclerosis involve expression of leukocyte and endothelial cell adhesion molecules. This is followed by the involvement of monocytes, macrophages and innumerable cytokines in the passage and accumulation of lipids. Late phases such as plaque rupture again involve macrophages and cytokine action, which stimulate the release of matrix metalloproteinases and other proteolytic enzymes. Additionally, vasoconstriction and spasm are in part induced by the action of pro-inflammatory cytokines, and an increased tendency toward coagulation results from actions of these same major pro-inflammatory mediators.

**Figure 2:**
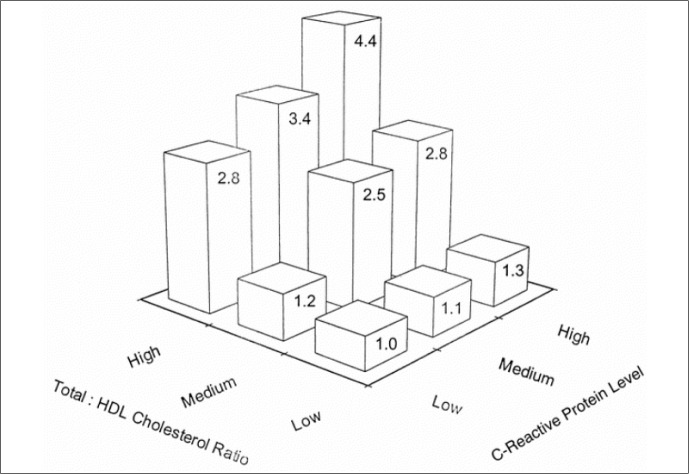
Relative risk of a future myocardial infarction as a function of baseline levels of C-reactive protein and the total cholesterol-to-HDL-C ratio. Ridker PM. Evaluating Novel Cardiovascular Risk Factors: Can We Better Predict Heart Attacks? Ann Intern Med 1999;130:933-37. Reproduced^11^ with permission.

Several epidemiological studies investigating prospective associations between markers of inflammation and cardiac events have repeatedly demonstrated the power of such markers to predict future events. For example, sialic acid, which can be considered an integrated marker of the levels of several acute phase proteins,^9^ predicts CHD, with men with highest quartile sialic acid levels having a relative risk for cardiovascular mortality^10^ of 2.4 (2.0 to 2.8) and women 2.6 (1.9 to 3.6). Several studies have demonstrated the capacity of C reactive protein (CRP), another acute phase protein, to predict events. As can be seen in data from the Physicians’ Health Study^11^ in Figure 2, after adjustment for multiple other CHD risk factors, those with higher levels of CRP, independent of the level of dyslipidemia, have greater risk of suffering an acute myocardial infarction. In diabetic patients, data from the ARIC Study^12^ demonstrate that diverse markers of inflammation, including an elevated leukocyte count, predict statistically significant increased risk. Those with leukocyte counts in the top quartile (> 7700 cells/mm^3^) at baseline had approximately double the risk of suffering an infarct over an ensuing mean 6.6 years of follow-up.

Investigation of mechanisms to explain these associations have centered on the three main direct mechanisms of CHD causation – the atherosclerotic plaque, increased vascular tone (endothelial dysfunction) with resulting vasoconstriction and vasospasm, and thrombosis. However, if inflammatory mechanisms underlie the metabolic syndrome, then they could also cause CHD via the abnormalities of this syndrome. In this regard, we have questioned whether the so-called "common soil" of CHD and diabetes could not be inflammatory in nature.^13^

### Inflammation and the Metabolic Syndrome: Cross-sectional Epidemiological Evidence

Stimulated by the line of reasoning presented by some of the above evidence, and the striking similarity of risk factors for CHD and diabetes, we initiated an investigation of the role of inflammatory mediators in metabolic syndrome and diabetes in the ARIC cohort.^14^ In initial investigation of several correlations (Table 2), we noted several small to moderate-sized, but highly statistically significant correlations between markers of inflammation and abnormalities of the metabolic syndrome. Associations were most notable with body mass index (BMI) and triglycerides, and were somewhat stronger with triglycerides than with other abnormalities.

**Table 2:** Correlation of metabolic and/or inflammatory risk factors in 12330* white and African-American men and women aged 45-64 participating in the baseline examination of the Atherosclerosis Risk in Communities (ARIC) Study

	WBC	ALB	FIB	TSSA	ORO	HAPT	a1AT
GLU	0.06	0.09	0.06	0.04	0.04	0.03	-.12
BMI	0.05	-.16	0.20	0.12	0.14	0.08	-.03
WHR	0.16	0.04	0.14	0.14	0.20	0.10	-.01
INS	0.10	0.01	0.14	0.26	0.18	0.11	.07
HDL-C	-.19	-.01	-.12	-.16	-.26	-.15	-.08
TG	0.16	0.08	0.02	0.40	0.22	0.12	-.02

*GLU = fasting serum glucose, BMI = body mass index, WHR = waist-to-hip ratio, INS = fasting serum insulin, HDL-C = high-density lipoprotein cholesterol, TG = triglycerides, WBC = white blood cell count, ALB = albumin, FIB = fibrinogen, TSSA = total serum sialic acid, ORO = orosomucoid, HAPT = haptoglobin, a1AT = a1-antitrypsin;*

* *Sialic acid, orosomucoid, haptoglobin and a1-antitrypsin were measured in a sub-sample of 610 individuals; All correlation coefficients > 0.02, and in the sub-sample > 0.08, are statistically significant (P < 0.05); Reproduced14 with permission.*

**Figure 3:**
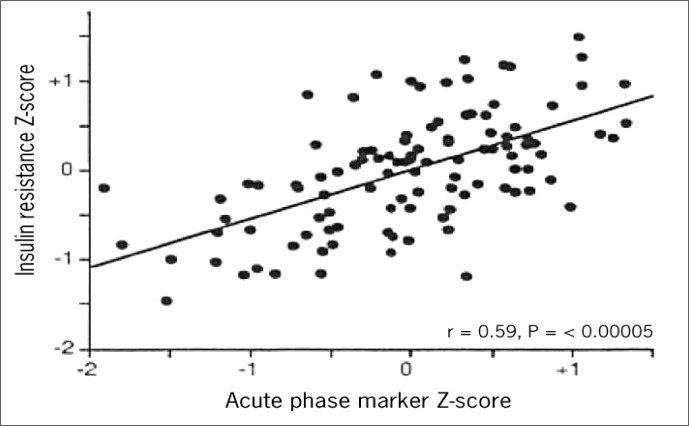
Association of the insulin resistance syndrome with the acute phase reaction. Yudkin JS, et al. C-reactive Protein in Healthy Subjects: Associations with Obesity, Insulin Resistance, and Endothelial Dysfunction. Arterioscler Throm Vasc Biol 1999;19:972-978. Reproduced^16^ with permission.

**Figure 4:**
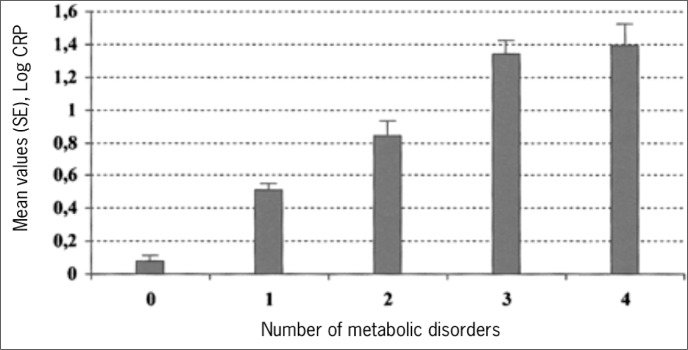
Association of levels of C-reactive Protein with the metabolic syndrome. Festa A, et al. Chronic subclinical inflammation as part of the insulin resistance syndrome. The Insulin Resistance Atherosclerosis Study (IRAS). Circulation 2000;102:42-47. Reproduced^17^ with permission.

Similar findings had been reported previously by Pickup and Crook,^15^ in a small clinical sample of patients with type 2 diabetes. Yudkin^16^ took this investigation one step further, in a relatively small sample, correlating a Z-score based on the levels of several metabolic syndrome abnormalities with a Z-score calculated on levels of various acute phase response proteins (Figure 3). A highly significant correlation (r = 0.59, P < 0.001) was found. A more recent demonstration of this association in a larger, free-living population comes from the Insulin Resistance Atherosclerosis Risk (IRAS) Study.^17^ Here (Figure 4), Festa et al. demonstrated a monotonic increase in CRP levels with the presence of an increasing number of metabolic syndrome abnormalities.

It is important to note that some of the largest associations with inflammatory markers are seen with BMI. In fact, adipocytes, especially in the obese, have been shown to produce a wide range of pro-inflammatory cytokines and other mediators, including leptin, TNFa, IL-6 and perhaps PAI-1.^18,19^ Mohamed-Ali et al.^20^ quantified post-prandial production of IL-6 in sub-cutaneous fat, with results suggesting that a large fraction of bodily production of this cytokine in the post-prandial period comes from adipocytes.

A logical question, given these findings, is: which come first, inflammatory markers or the metabolic syndrome? Demonstration of the presence of a chronic inflammatory state prior to the development of syndrome elements, as has been amply shown for CHD, would strengthen the hypothesis that a systemic inflammatory response contributes to the development of the syndrome.

### Inflammation and the Metabolic Syndrome: Prospective Epidemiological Evidence

To initiate investigation of the temporal relationship between inflammatory markers and syndrome abnormalities, we chose to study incident diabetes.^14^ We followed over 12000 men and women aged 45-64 years in the ARIC Study for approximately 8 years, during which over 1300 new cases of diabetes were ascertained. As can be seen in Table 3, those with elevations of 3 markers of inflammation, after adjustment for several risk factors for diabetes (Model 1), had significantly increased risk of developing diabetes. After further adjustment for BMI and WHR (Model 2), the association for albumin and fibrinogen was no longer present. Those, however, with leukocyte counts in the highest quartile had a 50% increased risk of developing diabetes. Moreover, as will be discussed below, the risk shown in Model 1 may be closer to the true situation, as Model 2 may be over-adjusted.

**Table 3:** Relative odds+ (comparing extreme quartiles) of developing diabetes mellitus for markers of inflammation in white and African-American men and women followed for an average of 7 years in the Atherosclerosis Risk in Communities Study, 1987-1998

Marker	Model 1		Model 2	
	OR	95% CI	OR	95% CI
White cells, 1000/mm^3^	1.9	1.6 to 2.3	1.5	1.3 to 1.8
Low serum albumin, g/l	1.3	1.0 to 1.5	0.98	0.80 to 1.2
Fibrinogen, g/l	1.2	1.0 to 1.5	0.93	0.77 to 1.1

*+ as determined in logistic regression; OR = Odds ratio; CI = confidence interval; Model 1: adjusted for age, gender, race and center, baseline glucose, family history of diabetes, physical activity and pack-years of cigarette smoking; Model 2: adjusted additionally for body mass index and waist-to-hip ratio; Adapted14 with permission.*

Sialic acid and three acute-phase proteins had previously been measured in a subset of approximately 600 of these individuals. Over an average of 4.9 years, 33 new cases of diabetes were ascertained in this subset. Comparing high with low levels of these markers at baseline with the development of diabetes in multiply adjusted analyses, we found that sialic acid and, to an even greater extent, the acute-phase protein orosomucoid predicted the development of diabetes (Table 4). Interestingly, associations were strongest over the first follow-up period.

**Table 4:** Relative odds+ of developing diabetes mellitus for those with values above the median for sialic acid and three acute phase proteins in 610 white and African-American men and women followed for an average of 4.6 years in the Atherosclerosis Risk in Communities Study, 1987 to 1996

Marker	Model 1		Model 2			
			All Cases		First 3 years++	
	OR	95% CI	OR	95% CI	OR	95% CI
Sialic acid	3.7	1.4 to 9.8	2.8	1.0 to 8.1	4.4	1.1 to 16.8
Orosomucoid	7.9	2.6 to 23.7	7.1	2.1 to 23.7	7.9	1.9 to 32.3
a_1_-antitrypsin	1.0	0.4 to 2.4	1.1	0.4 to 2.8	1.8	0.6 to 4.9
Haptoglobin	1.7	0.7 to 4.0	1.6	0.6 to 4.1	2.1	0.7 to 6.0

+ *As determined in logistic regression modeling;*

++ *Analyzing only cases detected at ARIC Visit 3, encompassing, approximately the first three years of followup; OR = odds ratio; 95% CI = 95% confidence interval; Model 1: adjusted for age, gender, ethnicity, atherosclerosis case-control status, fasting plasma glucose, family history of diabetes and smoking status; Model 2: adjusted additionally for body mass index and waist-to-hip ratio; Reproduced^14^ with permission.*

**Figure 5:**
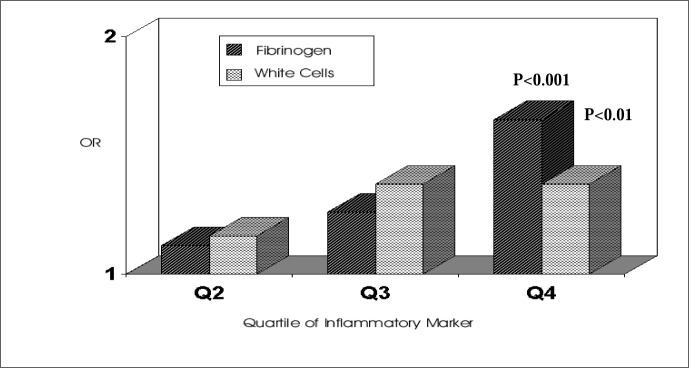
Relative-odds of having a major (> 90% ile) weight gain over 3 years of follow-up as a function of baseline levels of fibrinogen and white cell count. The Atherosclerosis Risk in Communities Study.

Given that obesity is a central element of the metabolic syndrome, we next investigated the possibility that elevation in inflammatory markers could also predict weight gain.^21^ A large weight gain was defined as one greater than the 90^th^ percentile for the cohort over the three-year period following baseline examination, or approximately 6 kg. The risk of having a large weight gain, after multiple adjustment, including baseline BMI, was again predicted by high levels of several inflammatory markers, most notably for fibrinogen (Figure 5). In fully adjusted analyses, those with baseline fibrinogen values in the highest quartile had 65% greater odds of having a large weight gain over the ensuing 3 years than those with values in the first quartile.

### The Innate Immune System

These findings led us to seek potential explanations. An attractive one has been offered by Pickup and Crook.^22^ These investigators, noting that Type 2 diabetes mellitus is characterized by an immuno-metabolic profile consistent with activation of the innate immune system, proposed that Type 2 diabetes might be a disease of this system.

The human immune system is based on both acquired (specific) and innate (relatively non-specific) immunity. Acquired immunity is based on cellular processing, over a period of days, of unique antigens with subsequent production of antibodies and other immune molecules specific to them. Innate immunity, on the other hand, is "hard wired", generating an immediate response to perceived threats to bodily integrity. Innate defense mechanisms include physical barriers, soluble factors such as the complement cascade, chemokines and cytokines, as well as white cells such as monocytes, macrophages and neutrophils, whose bactericidal action, although related to acquired immune mechanisms, is distinct from that of the T and B cells of acquired immunity.^23^

All of the components of the inflammatory response and of endothelial dysfunction which have been linked to cardiovascular disease, the metabolic syndrome and diabetes are, in one form or another, related to this innate immune system. Some components indicate the presence of activators of the system (infections such as Chlamydia and cytomegalovirus, cigarette smoking); others its molecular mediators (C reactive protein, serum amyloid A, sialic acid); and still others, downstream results of its activation (adhesion molecules such as ICAM, matrix metalloproteinases, and vasoconstriction).

The innate system, although phylogenetically more primitive than the acquired, has a fundamental role in human survival. Its defense mechanisms can be viewed as basically antimicrobial and hemostatic. They are orchestrated primarily by cytokines, polypeptides produced by many cells, particularly monocytes and macrophages, whose main function is intercellular signaling. Most pro-inflammatory cytokines originate in multiple cell types and have multiple target cells and functions, with autocrine (same cell), paracrine (neighboring cell) and epicrine (distant cell) actions. Among them, the pro-inflammatory cytokines^5^ tumor necrosis factor-a (TNF-a), interleukin-6 (IL-6) and leptin have received greatest attention as links in the disease processes cited above. Most importantly, activation of this system leads to fundamental changes in body metabolism.^24^

Given an acute infectious stress, the innate system immediately responds with the so-called acute-phase reaction, modulated by cytokines and characterized by a wide range of changes in behavioral function (somnolence), physiology (stimulation of the hypothalamic-pituitary-adrenal axis, increase in catecholamine secretion, leukocytosis), biochemistry (increase in hepatic lipogenesis and adipose tissue lipolysis), and nutritional function (anorexia).^25^ The new state achieved can be transitory in nature, if the precipitating stress is self-limited or treated with success. However, if the stressor persists, a chronic, semantically paradoxical "chronic acute-phase response" can result. Characteristics of this chronic state, being more recently noted, are less clear, though they apparently differ from those of the acute state at least in terms of behavioral and nutritional function.

Given the important complementary roles of innate immunity and the neuroendocrine system in response to stress, it is important to examine possible interfaces of these systems in the development of the metabolic syndrome. In this regard, Bjorntorp has recently summarized evidence that stress-related hypothalamic arousal, with associated neuroendocrine alterations, particularly perturbations of the hypothalamic-pituitary-adrenal axis, is a major pathway to the metabolic syndrome. Following this theory, daily life stresses would lead to dysregulation of feedback on this axis, with an altered cortisol biorhythm and inhibition of growth hormone and sex hormone production. Concomitantly, the sympathetic nervous system would be activated. The metabolic consequences suggested include central obesity and insulin resistance.^26,27^ In this regard, it is notable that pro-inflammatory cytokines are major stimulants of the hypothalamic-pituitary-adrenal axis, leading to increased cortisol secretion and inhibition of sex hormone production.^28^

**Figure 6:**
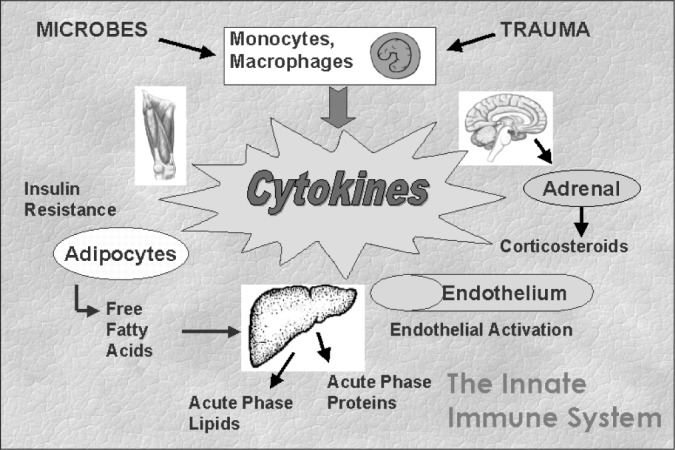
Major actions of cytokines in the innate immune system.

Figure 6 summarizes the systemic actions of relevant cytokines that could contribute to the pathogenesis of the metabolic syndrome, diabetes, and the common causality of diabetes and CHD. The theoretical basis of these interrelations was recently reviewed.^22^ Notable in the figure is that cytokine actions can directly result in insulin resistance, a basic mechanism in the pathogenesis of type 2 diabetes, ^29^ as well as producing the dyslipidemic condition typical of the syndrome.^30^ In addition, via endothelial activation, they could favor vasoconstriction and hemostasis, leading to the inclusion of chronic endothelial activation (endothelial dysfunction) as an integral part of the chronic systemic inflammatory response.

### An Integrated Picture: Innate Immunity, Neuroendocrine Modulation, and the Metabolic Syndrome

**Figure 7:**
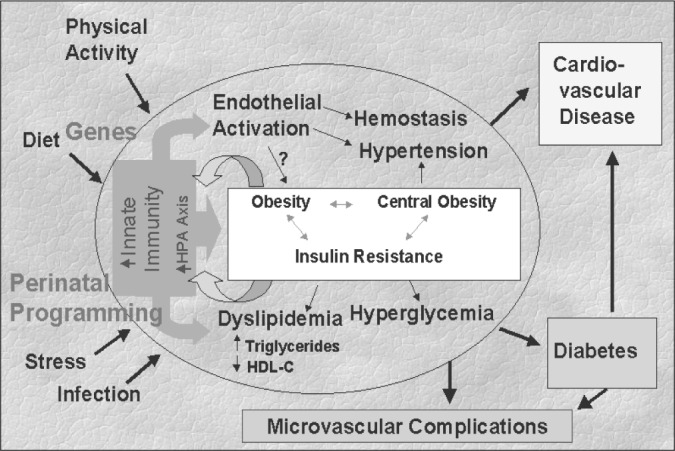
Conceptual framework for understanding the role of the innate immune system in the development of the metabolic syndrome and its complications.

Given the above, it is possible to propose that a chronic activation of the innate immune system underlies the metabolic syndrome, as well as the "common soil" of diabetes and cardiovascular disease. This is illustrated in Figure 7.

The innate immune system, conditioned by genetic factors and immuno-metabolic programming (altered gene expression profile) resulting from fetal and early life stressors, can be activated by a series of insults, including infection, physical trauma and emotional stress. Components of this system, via their effects on insulin resistance,^29^ and perhaps also through weight gain^21^ and central obesity, could be basic pathogenic mechanisms for the development and maintenance of the metabolic syndrome. Also, as previously mentioned, inflammatory cytokines induce elevation of triglycerides and reduction of HDL-C,^30^ especially when associated with obesity and can produce endothelial activation, with a resulting tendency toward vasoconstriction (hypertension) and hemostatic alterations.

On the other hand, obesity, once present, may reinforce innate immune hyperactivity, as adipose tissue is an important source of pro-inflammatory mediators such as TNF-a, IL-6, and leptin.^19^ Thus, the hyperproduction of so-called adipocytokines and other inflammatory agents, which could well be called obesitis, could maintain a state of chronic activation of the innate immune system, perpetuating the systemic inflammatory base of the syndrome. In addition, it is possible that insulin resistance, once installed, could also reduce insulin's inhibitory actions in the acute phase^31,32^ also favoring maintenance of a chronic state of inflammation.

Thus, as illustrated in Figure 7, depending on genetic predisposition, on the results of early-life stresses incorporated into expression of this genetic background, and on current lifestyle and environmental factors, this chronic activation of the innate immune system could lead to the development of diabetes and CHD. In this regard, the extent to which, and the pathogenic pathways by which diabetes may independently contribute to cardiovascular disease remain to be established. One possibility is that the glycosylation and advanced glycosylation end product (AGEs), caused by hyperglycemia via the resulting oxidative stress,^33^ further induce pro-inflammatory cytokines and their deleterious consequences.

## CONCLUSION

Components of the metabolic syndrome aggregate in free-living populations. The prospective associations of several inflammatory markers with diabetes mellitus and weight gain, together with cross-sectional data from diverse populations showing correlations of inflammatory markers with metabolic syndrome components, separately and in aggregate, provide an empirical basis for the hypothesis that a chronic, mild inflammatory state is not only subjacent to cardiovascular disease, but also to this syndrome. Better understanding of the role of the innate immune system in the pathophysiology of obesity, diabetes, the metabolic syndrome and cardiovascular disease as well as of the causes of chronic activation of this system may lead to important advances in the prediction and management of these chronic diseases.
